# Intelligent anti-jamming communication technology with electromagnetic spectrum feature cognition

**DOI:** 10.1371/journal.pone.0319953

**Published:** 2025-04-24

**Authors:** Hui Zhao, Guobin Zhao, Xichun Wang, Zhonghui Zhang, Xianchao Xun

**Affiliations:** 1 College of Aviation Foundation, Aviation University Air Force, Jilin, China; 2 Physics Group, Jihua First Senior High School, Jilin, China; 3 High School Physics Group, Jilin Songhua River Middle School, Jilin, China; Parul University, INDIA

## Abstract

Against the backdrop of the rapid development of wireless communication technology, the complex signal interference issues in the electromagnetic spectrum environment have become a key factor affecting the quality and reliability of signal transmission. Existing solutions, such as traditional interference suppression techniques that rely on static spectrum allocation and fixed interference patterns, are no longer able to adapt to the rapidly changing electromagnetic environment and face computational complexity challenges when processing large amounts of real-time data. This study proposes an intelligent anti-interference algorithm that combines deep neural networks and game theory, and constructs a model based on near-end strategy optimization. By extracting and processing signal features through deep neural networks, and dynamically adjusting communication strategies with near-end optimization, the model effectively addresses the recognition and prediction of signal transmission feature parameters in target communication systems, generates interference signals with the same feature parameters, and achieves effective interference suppression. Experiments show that the proposed model achieves an accuracy rate of 95.23% in identifying interference signals and an anti-interference accuracy rate of 85.47%, significantly outperforming random forest and deep Q-network models. The study not only clarifies the limitations of existing solutions but also precisely defines the goals of the new model, which are to reduce error rates and improve adaptability in dynamic environments. The results further explain the significance of the used metrics and test conditions, providing new means and strategies for the development of anti-interference communication technology, especially in dealing with new complex electromagnetic spectrum interference.

## 1. Introduction

Currently, the rapid development of wireless communication technology has led to its widespread application in various fields. However, there are many complex signal interferences in the electromagnetic spectrum environment. In modern communication systems, interference and noise are key factors that affect the quality and reliability of signal transmission. Types of interference typically include frequency overlap, intentional or unintentional signal interference, and background noise [1]. For example, in dense urban environments, multiple wireless devices may operate on the same frequencies, resulting in frequency overlap and mutual interference [2]. In military communications, hostile forces may deliberately transmit jamming signals to weaken or block communication links [3]. These different types of signals pose many challenges to anti-interference capabilities and also cause serious interference problems, posing enormous challenges to communication systems. Infinite communication technology not only uses dedicated frequency channels for aviation radio navigation, distress search and rescue, radio astronomy and other services, but also shares signal radio services. The reason why they can be shared is mainly due to the three-dimensional characteristics of the electromagnetic spectrum in the spatial, temporal and frequency domains. When multiple frequency weapons and equipment are densely deployed, electromagnetic waves undergo dynamic changes between time and space in the spatial domain, and are densely interlaced in the frequency domain, resulting in overlapping problems between the three domains. This can cause the electromagnetic channels of spectrum equipment to intersect with each other, causing self interference, mutual interference, and easy interference [4]. Conventional methods have specific limitations in dealing with these interferences. For example, some conventional interference suppression techniques rely on static spectrum allocations and fixed interference patterns, which cannot be adapted to the rapidly changing electromagnetic environment [5]. Computational complexity is also an issue because as the complexity of a communication system increases, so does the need to process large amounts of data in real time, which places a higher demand on computational resources. In addition, traditional methods may lack the flexibility to adapt to new interference sources and unknown attack strategies [6]. Therefore, more advanced intelligent anti-jamming techniques are needed to overcome the complex signal jamming problem.

In this context, to improve the reliability of communication systems, this study proposes an intelligent anti-interference algorithm that combines Deep Neural Networks (DNN) and game theory. However, optimization based deep learning algorithms have limitations when facing complex interference, so this paper proposes an intelligent anti-interference algorithm with Near End Strategy Optimization (NSO). NSO adapts to real-time changing spectral environments by dynamically adjusting communication policies, while DNN can be used to identify and predict interference patterns for more accurate interference suppression. The proposed method utilizes the framework of Markov Decision Process (MDP) to optimize the strategy of communication systems, and then constructs an NSO based on the optimization strategy, making it more flexible and adaptable in the face of intelligent interference. The deep learning algorithm of ensemble game theory and NSO proposed in the research are expected to provide new means and strategies for anti-interference communication.

The study proposes an intelligent anti-jamming algorithm that combines DNN and game theory, and constructs a model based on NSO. The algorithm can adapt to the real-time changing spectrum environment and improve the reliability and anti-jamming capability of the communication system by dynamically adjusting the communication strategy. By identifying and predicting the set of characteristic parameters of the signals transmitted by the target communication system, it generates and transmits jamming signals with the same characteristic parameters to effectively jam the target communication system. In addition, the jamming model is able to adaptively switch according to the electromagnetic spectrum characteristics to achieve timely and effective jamming. The model performs well in complex electromagnetic environments, can effectively manage and cope with external interference, and improves the stability and security of the communication system. The proposed method provides new means and strategies for anti-jamming communications, especially in combating new complex electromagnetic spectrum interference.

The research is divided into four parts. The first part summarizes the research of domestic and foreign scholars on electromagnetic spectrum and communication anti-interference methods, and analyzes their research results; The second part is to construct an anti-interference model based on deep learning and NSO algorithm; The third part is to evaluate the model through performance testing and application analysis; The fourth part is to summarize the experimental results, point out the shortcomings of this study, and propose future research directions.

## 2. Related works

Nowadays, with the rapid development of wireless communication technology, there is an increasing amount of research on intelligent anti-interference technology. B. Simran. Charu Charu et al. proposed a Carrier Suppression Returns to Zero (CSRZ) method to address the adverse effects of atmospheric turbulence such as rain, fog, and haze on the performance of Free Space Optical Communications (FSO) systems. CSRZ has a significant effect when facing interference [7]. H. Karam et al. have developed a concept of using Time Reversal (TR) cavities to accurately locate electromagnetic interference sources. This method applies the entropy criterion to obtain focused time slices, which is economical and efficient [8]. A. de Jesus Torres provided a physically meaningful model for electromagnetic interference as a baseline for evaluating auxiliary communication. This model used Reconfigurable Intelligent Surfaces (RIS) to assist wireless communication systems, where each component of the RIS scattered the input signal with a controllable phase shift without increasing its power, which was beneficial for baseline evaluation [9]. J. Paś et al. conducted electromagnetic compatibility tests on lighting modules with converters using ESCI3 electromagnetic interference receivers and artificial networks to address electromagnetic interference in railway areas. This scheme could minimize the impact of electromagnetic interference on system functionality to the greatest extent possible [4]. T. N. Nguyen et al. proposed a method combining full duplex transmission and intelligent reflective surfaces to address the issues of self interference and hardware damage in wireless communication systems. This method could adjust the transmission power and the number of reflective elements according to actual needs to improve performance and energy saving [10].

A. Li et al. proposed a method of manipulating interference signals using symbol level precoding techniques to form beneficial superposition at the receiving end. This method could improve the performance of multi antenna wireless communication systems by optimizing the precoding design [ 11]. Y. Kawamoto et al. proposed a frequency resource allocation method that considered inter beam interference to address the issue of lack of flexibility in resource allocation. This method extended the traditional flexibility analysis model to more accurately quantify flexibility, and the effectiveness of the proposed method under changes in satellite communication system status was validated [12]. A. Al-Jumaily proposed a frequency resource allocation method that considered inter beam interference. By extending the traditional flexibility analysis model, it could more accurately quantify flexibility, and the effectiveness of the proposed method had been verified under changes in satellite communication system states [13]. S. A. Gbadamosi built an adaptive interference avoidance and mode selection framework, which utilized a two-stage resource sharing algorithm to reduce system computational complexity while maximizing network overall speed and reducing signaling overhead compared to other algorithms [14]. T. N. Nguyen et al. proposed the application of full duplex technology and partial relay selection methods for information transmission in satellite ground networks. This method effectively improved the reliability and reduced complexity of the system by increasing the number of relays or transmission power, while reducing the probability of interruption [15]. A summary of the relevant literature is shown in Table 1.

**Table 1:** Comparative analysis of existing methods.

Research content	Research methodology	Key results	Advantages	Disadvantages	References
Multi-beam free-space optical communication performance analysis	CSRZ	Improves the performance of FSO systems in the presence of interference	Significant performance improvement	Complex modulation and demodulation techniques are required	[7]
Electromagnetic interference source localisation	Time-reversal cavity	Accurate localisation of electromagnetic interference sources	Highly accurate, cost-effective	Sensitive to environmental changes	[8]
Electromagnetic interference in RIS-assisted communications	Physical significance model	Provides a baseline model for evaluating assisted communications	Helps to understand the application of RIS in communications	Range of applications is one-sided	[9]
EMI analysis of LED lighting modules	Reliability- operational analysis	Analysed EMI in LED lighting modules	Provided detailed EMI analysis	Not all sources of interference were considered	[4]
Intelligent reflective surfaces in full-duplex communication systems	Full-duplex transmission with intelligent reflective surfaces	A solution to self-interference and hardware impairments is proposed	Improved performance and energy savings	Complex hardware support is required	[10]
Symbol- level precoding techniques	Symbol-level precoding	Improves the performance of multi-antenna wireless communication system	Optimises precoding design	Requires complex signal processing	[11]
Resource allocation in satellite communication systems	Resource allocation method	A resource allocation method considering inter-beam interference is proposed	Resource allocation flexibility is improved	Complex computation is required	[12]
5G coexistence and interference signal assessment	Flexibility analysis model	A methodology for assessing 5G coexistence and interference signals is proposed	Improves the accuracy of flexibility quantification	Needs more experimental validation	[13]
Interference avoidance and mode selection in 5G-IIoT networks	Two-stage resource sharing algorithm	Reduces system computational complexity	Maximises overall network speed	Requires complex algorithmic support	[14]
Full-duplex relay selection in satellite terrestrial networks	Full-duplex technique and partial relay selection	Improves system reliability and reduces complexity	Increases relay or transmission power	Requires complex relay selection strategies	[15]

Combining the above, it can be seen that the adaptive framework and electromagnetic spectrum processing and the interference of beams proposed by the researchers have a wide range of application prospects in malicious spectrum interference, which provides an important theoretical basis for the development of communication security. However, there are currently few studies that combine deep learning algorithms with alliance game techniques, and there is also a lack of analysis on strategy optimization. In addition, existing studies have more often used frequency hopping techniques to defend against interference, but these methods perform poorly in the face of rapidly changing electromagnetic environments. They are unable to adapt to new interference sources in real time, leading to a decrease in interference immunity. And computational complexity is also an issue, as traditional methods usually require a large amount of computational resources, limiting their effectiveness in real-time applications. Therefore, this study utilizes alliance game theory to optimize the DNN’s anti-interference algorithm and constructs an NSO. The innovation of this study lies in optimizing DNN through alliance game technology, aiming to resist new complex interference in the electromagnetic spectrum and provide more technical support for the security of communication signals.

## 3. Intelligent anti-interference communication algorithm based on feature cognition

### 3.1. Intelligent anti-interference algorithm based on DNN combined with game theory

#### 3.1.1. Deep neural network based anti-jamming communication algorithm.

**Fig 1:**
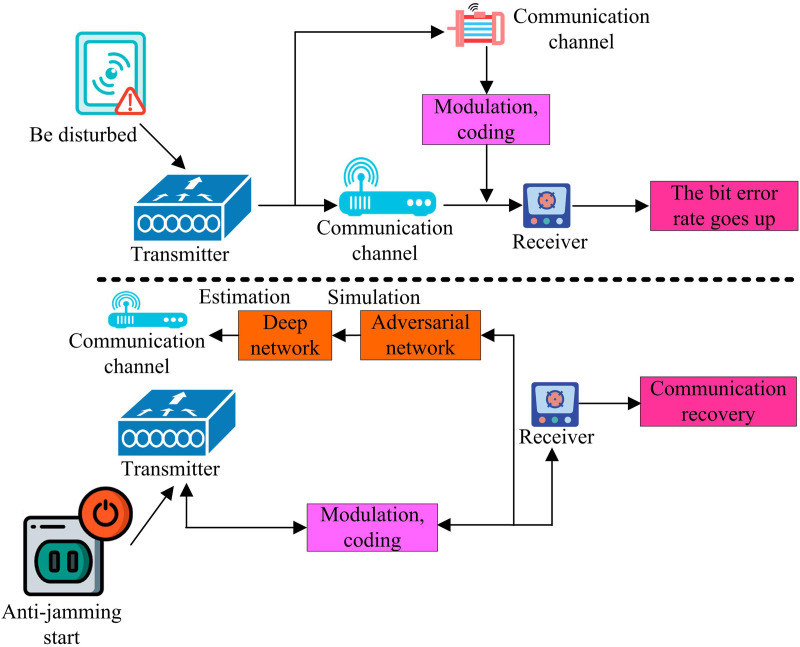
Anti-jamming communication process.

Electromagnetic spectrum sensing involves signal detection, the core of which lies in identifying signal sources in a specific frequency band by detecting signals in the electromagnetic spectrum. However, signals in the spectral environment are usually affected by a variety of factors, which puts higher demands on the performance of anti-jamming algorithms [16,17]. The traditional anti-jamming techniques are not able to cope with the new types of complex interferences appearing in the electromagnetic spectrum. Therefore, the study proposes to optimise the anti-jamming algorithms using deep learning neural networks combined with game theory. The anti-jamming communication flow based on DNN is detailed in Fig 1.

In Fig 1, two different states are shown: normal communication, interference, and activation of anti-interference. When the communication system is affected by interference, the transmitter in the system sends interference signals through the signal path [18,19]. When the communication system detects the characteristics of interference signals, these signals will be modulated, encoded, and specially designed by the interfering device, and then sent to the receiving end through wireless channels. When the receiving end processes these signals, the communication error rate will correspondingly increase. When interference is introduced into the communication system, the bit error rate at the receiving end increases. Therefore, the study uses DNN to extract and process signal features. Firstly, the received signal is normalised to scale the signal data to a fixed range and the time and frequency domain features of the signal are extracted using a convolutional layer. The convolution operation captures the local features of the signal, which is expressed as shown in equation (1).


$$(f*g)(t)=\int_{-\infty }^{\infty }f(\tau )g(t-\tau )d\tau$$ (1)

In equation (1), *f* denotes the input signal. *g* denotes the convolution kernel; *t* denotes time;  ∗  denotes the convolution operation; f(τ) denotes the value of the convolution kernel at position *τ*. And g(t−τ) denotes the value of the input signal at time *t* minus *τ*. Meanwhile, each convolutional layer is accompanied by a maximum pooling layer to reduce the number of parameters and extract key information. After feature extraction, the features are integrated using a fully connected layer and the classification or regression task is performed and the final classification result is output using a softmax layer. In this case, the generator of DNN can be expressed as equation (2).


$$L_{G}=\underset{\theta_{G}}{\min}E_{z-p_{z}}\left[\log\left(1-D\left(G\left(z\right)\right)\right)\right]$$ (2)

In equation (2), *G* represents the generator; *D* represents the discriminator; $p_{z}$ represents the prior distribution; $\theta_{G}$ represents the network parameters of the generator; *E* represents the expected value. The discriminator of the DNN can be expressed as equation (3).


$$L_{D}=\underset{\theta_{D}}{\max}\left(E_{x-p_{data}}\left[\log\left(D\left(x\right)\right)\right]+E_{z∼p_{y}}\left[\log\left(1-D\left(G\left(z\right)\right)\right)\right]\right)$$ (3)

In equation (3), $\theta_{D}$ represents the network parameters of the discriminator. The generator obtains effective information from the previous stage through a DNN to assist in channel generation, so the loss function of the generator can be expressed as equation (4).


$$L_{G}=\underset{\theta_{G}}{\min}E_{z∼p_{z}}\left[\log\left(1-D_{\theta_{D}}\left(x,z,\overset{\wedge }{\text{H}}\right)\right)\right]$$ (4)

In equation (4), *x* represents the signal after encoding and modulation; *z* represents random noise; $\overset{\wedge }{\text{H}}$ represents the channel estimation result. The task of the discriminator is to determine whether the received samples come from the simulated channel of the generator or the real channel based on their distribution, to make a binary judgment of authenticity. So the loss function of the discriminator can be expressed as equation (5).


$$L_{D}=\underset{\theta_{D}}{\max}\left(E_{z∼p_{z}}\left[\log\left(1-D_{\theta_{D}}\left(x,z,\overset{\wedge }{\text{H}}\right)\right)\right]+E_{y∼p_{y}}\left[\log\left(D_{\theta_{D}}\left(x,z,\overset{\wedge }{\text{H}}\right)\right)\right]\right)$$ (5)

By iteratively training the generator and discriminator, their ability to complete their respective tasks gradually improves. The final generator’s simulation of the real channel becomes increasingly accurate, causing the discriminator to become ineffective.

#### 3.1.2. Optimisation strategies for deep neural networks combined with game theory.

**Fig 2:**
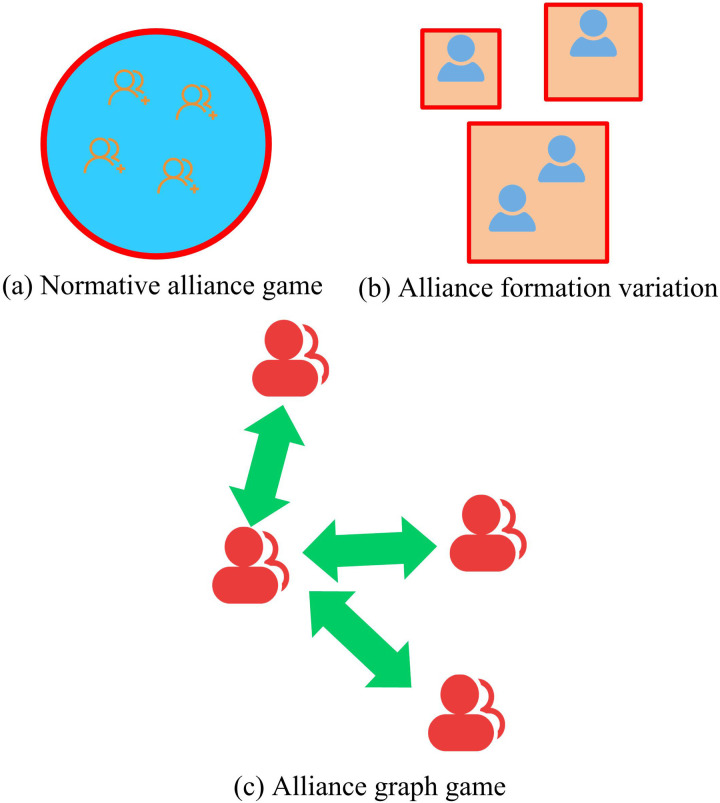
The classification method of the alliance game.

In order to better combat external interference in the frequency domain and using coalition games to classify the signals that interfere with the electromagnetic spectrum. In this case, the coalition game is classified as shown in Fig 2.

In Fig 2, game theory provides a complete analysis framework and appropriate approach for interference signals in the electromagnetic spectrum. Through the control of alliance games, the interaction between multiple decision-makers can be further deepened and strengthened. In the decision-making of intelligent communication anti-interference, game theory is generally used to model the data transmission behavior of both parties in adversarial communication [20]. The study uses coalitional games to model cooperative and competitive relationships between multiple decision makers. By constructing a payoff matrix, the payoff of each decision maker under different strategies is analysed and the optimal cooperation strategy is found. At the same time, the game-theoretic control behaviour is used to provide a theoretical analysis of the confrontation strategies between the two parties in the electromagnetic spectrum. When the game is completed it is possible to foresee the form of confrontation afterwards. The game of coalition formation requires decision making through co-operation between decision makers, and decision makers need to abide by the agreed decisions. In alliance games, a key issue is how to fairly distribute the total benefits obtained by the alliance, which can be expressed as equation (6).


$$\Phi_{i}\left(N,v\right)=\sum S\subseteq N\backslash \left\{i\right\}\frac{\left|S\right|!\left(\left|N\right|-\left|S\right|-1\right)!}{\left(\left|N\right|-1\right)!}\left[v\left(S\cup \right)\left\{i\right\}-v\left(S\right)\right]$$ (6)

In equation (6), $\Phi_{i}$ represents the benefit value that decision-makers should receive in the alliance; *i* represents the decision-maker coefficient; *N* represents the decision-maker set; *v* represents the feature set function; *S* represents a subset of the decision-maker set. In the game of alliance formation, the stability of the alliance needs to be considered. This study introduces the core concept of satisfying the set of income distribution, which can be expressed as equation (7).


$$C\left(v\right)=\left\{x\in R^{\left|N\right|} |x\left(N\right)=v\left(N\right),S\subseteq N,x\left(s\right)\geq v\left(s\right)\right\}$$ (7)

In equation (7), $C\left(v\right)$ represents the core set; *x* represents the profit distribution vector; *R* represents a constant. In alliance games, the relationships between decision-makers can be modeled as a graph, where nodes represent decision-makers and edges represent their cooperative or competitive relationships. The concept of degree centrality in graph theory is used to measure the importance of a node in a network, and degree centrality is represented by equation (8).


$$DC_{i}=\frac{\deg\left(i\right)}{\left|N\right|-1}$$ (8)

In equation (8), $DC_{i}$ represents importance; $\deg\left(i\right)$ represents the degree of node *i*, which is the number of nodes directly connected to it. The clustering coefficient can represent the degree of connection between nodes, expressed by equation (9).


$$CC_{i}=\frac{2T_{i}}{\deg\left(i\right)\deg\left(i\right)-1}$$ (9)

In equation (9), $T_{i}$ represents the number of triangles passing through node *i*. In adversarial games, the optimal strategy usually refers to Nash equilibrium. In Nash equilibrium, no player can achieve higher returns by unilaterally changing their strategy. However, the environmental problem of SNR ratio in the game process cannot be ignored in the study. In the Nash equilibrium, the party except the decision maker should adjust according to the interference noise and interference power. Game theoretic dynamics in real-time communication systems are better captured with a payoff matrix always with interference parameters.The parameter can be expressed as Equation (10).


$$P\left(s_{i},s_{i-1}\right)=\log_{2}\left(1+\frac{S}{I+N}\right)$$ (10)

**Fig 3:**
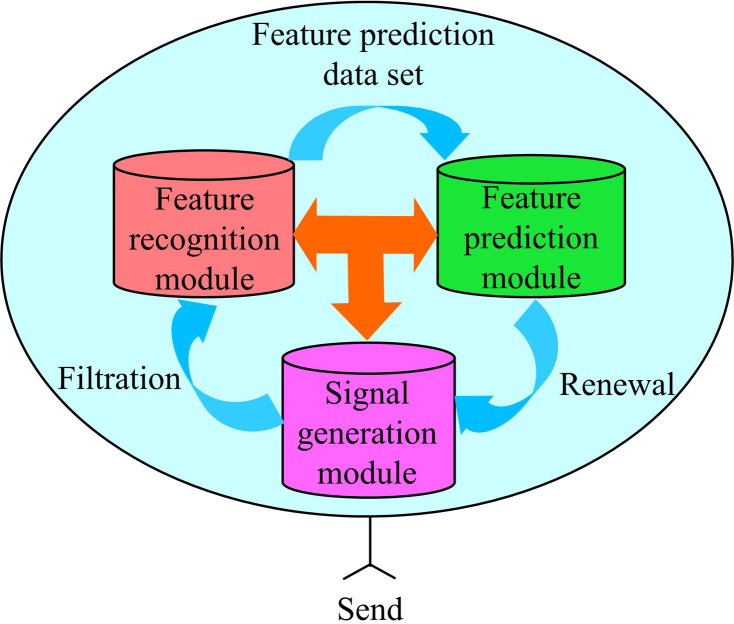
Intelligent interference system of degree of DNN control.

In equation (10), $s_{i}$ represents the decision-maker’s strategy; $s_{-i}$ represents the set of strategies other than the decision-maker; In equation (10), *P* represents the signal interference parameter, *I* represents the signal interference frequency, and *S* represents the signal power, In the training process of the model, a large number of negative samples can be added for training, so that the model can better distinguish the target. During signal monitoring, the predicted result determines whether the signal can be output as the result. Code error rate can be controlled by proper adjustment of the threshold. A higher threshold can reduce the bit error rate, but will increase the under reporting rate, a lower threshold increases the bit error rate, but then the corresponding false reporting rate will increase. The intelligent interference system controlled by DNN is shown in Fig 3.

In Fig 3, the signal feature prediction module and the signal feature recognition module are two key components. The signal feature prediction module is responsible for predicting the characteristics of interference signals and providing prior knowledge to the system; The signal feature recognition module analyzes the received signal in real time and identifies some hidden sources of interference [21]. The communication and connection between the two modules enable the system to quickly respond to interference signals. DNN continuously optimize the parameters of the interference signal generation module by learning historical data and real-time signal features, generating effective interference signals to counteract external interference. The system also includes a filtering and updating mechanism [22,23]. This mechanism ensures the real-time and effectiveness of interference signals, and improves the system’s anti-interference ability. The intelligent interference communication system guides the decision-making process of intelligent agents through alliance game theory, optimizes the generation of interference signals using deep learning technology, and achieves effective management and response to external interference through dynamic filtering and updating mechanisms. The DNN also controls the signal by reducing the bit error rate. The convolutional layer in DNN has strong signal recognition ability, which can extract the features of interference signals. After the filtering of the convolution layer, a few interference signals enter the communication channel, and the noise width becomes smaller and the noise density increases, so the error rate decreases. The mathematical modeling of DNN can be expressed as equation (11), which to some extent reflects the error correction ability of the convolution layer.


$$BER=Q\left(\frac{\sqrt{2R}}{N_{o}}\right)$$ (11)

In equation (11), *Q* represents the tail probability of a standard normal distribution, which is used to calculate the probability of exceeding a certain threshold value. *R* is the encoding rate, which refers to the ratio of the number of information bits transmitted after encoding to the total number of transmitted bits, reflecting the encoding efficiency. In this model, the bit error rate BERcan be effectively reduced by adjusting the encoding rate *R* and reducing the noise density $N_{o}$. When using DNN for feature extraction and interference processing, optimizing the network structure and training process can help improve the signal quality, thus indirectly reducing $N_{o}$ or increasing *R*, and finally achieving the purpose of reducing the bit error rate.

**Fig 4:**
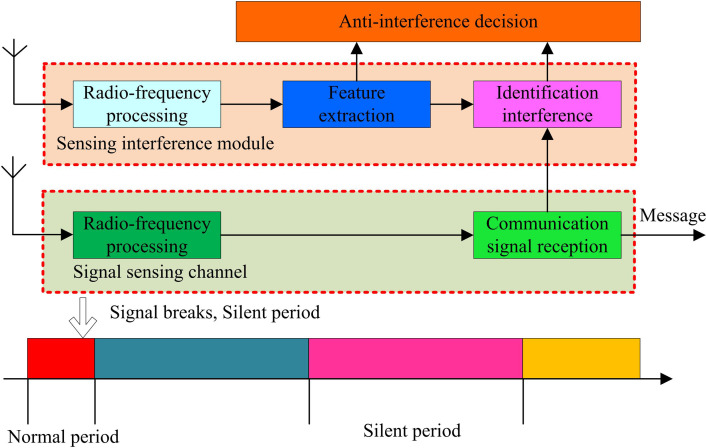
Game theory system of timing diagram of the anti-interference pattern.

The anti-interference timing diagram can be represented as Fig 4.

In Fig 4, the system first monitors abnormal signals in the electromagnetic spectrum through the interference sensing module. This module utilizes radio frequency processing technology to conduct preliminary analysis on the received communication signals, to identify possible sources of interference. Subsequently, key information that contributes to interference identification is extracted. Once interference is identified, the system will make decisions based on communication parameters and the current communication status. During normal communication, the system maintains the continuity of communication; When interference is detected, the system will enter a silent period to avoid the impact of interference on communication quality [24]. During the quiet period, the system not only avoids direct interference effects, but also provides a time window for interference identification and decision-making. Deep learning algorithms can learn interference patterns from historical data, while game theory provides theoretical support for agents to make optimal decisions in interference environments. Through this combination, intelligent jamming communication systems can quickly respond when interference occurs and generate effective jamming countermeasures. This strategy not only includes direct confrontation with interference signals, but also dynamic adjustment of communication parameters to optimize communication performance.

### 3.2. Anti-jamming communication model based on near-end policy optimisation

**Fig 5:**
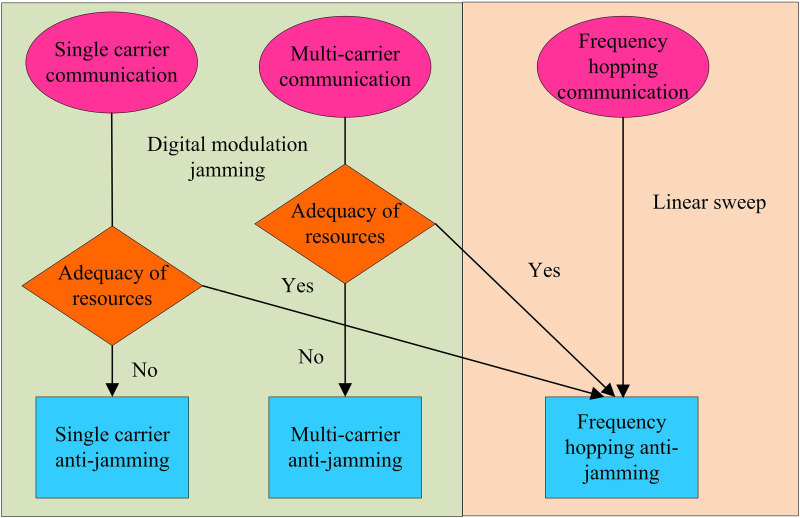
Optimized based on the proximal strategy decision logic.

In order to improve the effectiveness of the previously proposed anti-jamming communication algorithm, the study further introduces NSO to design an intelligent anti-jamming communication model. Based on the theoretical framework of Markov decision process, NSO is used to improve the robustness and adaptability of the communication system in the face of intelligent interference. The decision-making implementation logic of anti-jamming is specifically shown in Fig 5.

Fig 5 shows the adaptability and selection logic of different communication technologies in the face of interference. Single carrier communication is known for its simple structure, but it may be vulnerable to aiming interference. Multi carrier communication increases the system’s anti-interference capability by distributing signals across multiple subcarriers. Frequency hopping communication improves the ability to resist aiming interference by rapidly changing the communication frequency, making it difficult for jammers to continue interfering. Digital modulation technology can adjust the frequency characteristics of signals to adapt to different interference environments [25,26]. When spectrum resources are sufficient, multi carrier and frequency hopping communication can more effectively utilize these resources to enhance anti-interference capabilities. In the anti-jamming communication strategy, the decision logic update based on the NSO algorithm is represented in equation (12).


$$\pi \theta_{new}\left(a |s\right)\leftarrow \frac{\pi \theta_{old}\left(a |s\right)}{clip\left(\frac{\pi \theta_{new}\left(a |s\right)}{\pi \theta_{old}\left(a |s\right)},1-\varepsilon ,1+\varepsilon \right)}$$ (12)

In equation (12), $\pi \theta_{new}$ and $\pi \theta_{old}$ denote the old and new strategies, respectively. *a* denotes the action, *s* denotes the decision. And *ε* denotes the algorithm parameters, respectively. The objective function of the NSO algorithm is shown in equation (13).


$$L^{CLIP}=-E_{t}\left[\min\left(r_{t}\left(\theta \right)A_{\theta }\left(s_{t},a_{t}\right),clip\left(rt\left(\theta \right),1-\varepsilon ,1+\varepsilon \right)A_{\theta }\left(s_{t},a_{t}\right)\right)\right]$$ (13)

In equation (13), $L^{CLIP}$denotes the objective function of the algorithm. *E*denotes the expected value. $r_{t}$denotes the limiting probability, $A_{\theta }\left(s_{t},a_{t}\right)$denotes the dominance function of the algorithm and *θ*denotes the parameters of the objective function. The update of the value function for interference states in the electromagnetic spectrum can be represented by equation (14).


$$V\theta_{v}\left(s_{t}\right)\leftarrow V\theta_{v}\left(s_{t}\right)+\alpha \left|R_{t}-V\theta_{v}\left(s_{t}\right)\right|$$ (14)

In equation (14), $V\theta_{v}\left(s_{t}\right)$ represents the value estimation function of the algorithm; $\left(s_{t}\right)$ represents the state of the algorithm when faced with interference in the spectrum; $R_{t}$ represents the reward obtained by the agent from the environment; *α* represents the learning efficiency. The update of the action value function in the algorithm is represented by equation (15).


$$Q_{\theta_{q}}\left(s_{t},a_{t}\right)\leftarrow Q_{\theta_{q}}\left(s_{t},a_{t}\right)+\alpha_{q}\left|r_{t}+\gamma V_{\theta_{v}}\left(s_{t+1}\right)-Q_{\theta_{q}}\left(s_{t},a_{t}\right)\right|$$ (15)

In equation (15), $Q_{\theta_{q}}\left(s_{t},a_{t}\right)$ represents the action value function; $\alpha_{q}$ represents the learning factor; *γ* represents the discount factor. The interference impact assessment can be represented by equation (16).


$$I_{impact}=\frac{R_{clean}-R_{\mathrm{int}erf}}{R_{clean}}$$ (16)

In equation (16), $R_{clean}$ represents the return when there is no interference; $R_{\mathrm{int}erf}$ represents the return when there is interference. Spectrum resource allocation optimization can be represented by equation (17).


$$\max_{p}\sum_{i=1}^{N}Rate_{i}\left(P\right),s.t,\sum_{i=1}^{N}P\leq P_{total}$$ (17)

**Fig 6:**
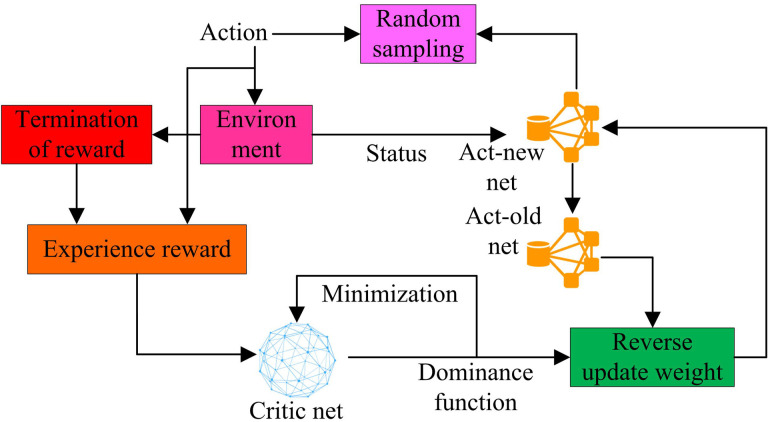
Box of the NSO.

In equation (17), *N* represents the number of spectral carriers; $Rate_{i}\left(P\right)$ represents the transmission rate of the *i* -th subcarrier; *P* represents the power allocated to the subcarrier; *i* represents the spectral coefficient; $P_{total}$ represents the total power; s.t represents the constraint condition. The study proposes to use the NSO algorithm to solve to obtain the optimal joint anti-jamming method of the system, so as to improve the convergence speed of the algorithm and shorten the convergence time to reach the optimal strategy, the specific implementation framework is shown in Fig 6.

In Fig 6, the core of the NSO framework includes Actor network and Critic network, which work together to achieve policy improvement [27,28]. The Actor network is responsible for generating actions, which are sampled from the current state of the policy. In the framework, the Actor new network represents the updated policy, while the Actor old network represents the old policy. The NSO guides the updating of network parameters by comparing the ratio of action probabilities between new and old policies, ensuring that the magnitude of policy updates remains within a reasonable range. The Critic network evaluates the performance of the current strategy by calculating the dominance function to measure the value of each action relative to the average strategy. A key step in the NSO framework is to reverse update the weights, which is achieved by minimizing the loss function. The specific calculation formula is shown in equation (18).


$$L(\theta )=-E_{\tau ∼\pi \theta }[\sum_{t=0}^{\infty }\gamma^{t}R(s_{t},a_{t})]$$ (18)

**Fig 7:**
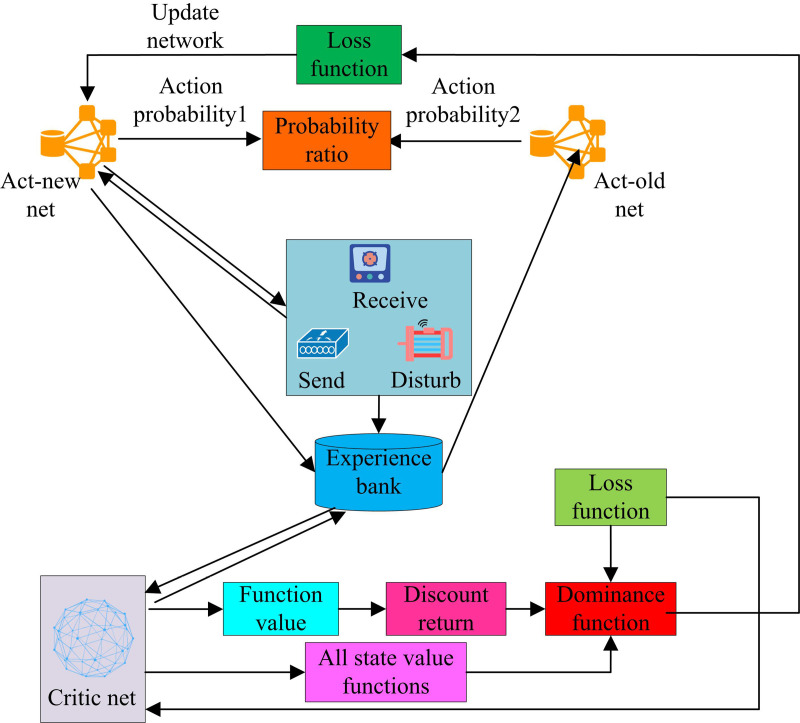
Decision model based on the NSO.

In equation (18), L(θ) represents the loss function, which is used to measure the difference between strategy πθ and the optimization objective. *E* stands for expected value; *γ* is the discount factor. $R(s_{t},a_{t})$ represents the instant reward obtained by time step *t*. Policy loss ensures the closeness of policy updates, while value loss helps Critic networks evaluate states more accurately [29,30]. After the intelligent agent performs actions in the environment and receives rewards, this information is stored in the experience pool, which is then used to update the Actor and Critic networks. If the intelligent agent reaches the termination state, the current iteration process ends, and the algorithm evaluates the strategy and updates the network parameters based on the collected experience. So the decision model based on NSO is shown in Fig 7.

In Fig 7, the model consists of an Actor network and a Critic network, which learn the optimal anti-interference strategy through interaction. Actor networks are responsible for generating action probabilities, which are the probabilities of selecting the best action in a given state. The probability of action represents the different actions that may be taken in different states. The Actor network is updated through a loss function that reflects the difference between the current policy and the optimization objective. The Critic network evaluates the performance of the current strategy and updates the network parameters through a loss function. It calculates the value function of the state and the advantage function of the action, which help evaluate the expected return of taking a specific action in a specific state. The experience library is used to store experience, which comes from many state situations obtained from environmental interactions and information about the next state. The entire model update process involves iterative optimization of Actor and Critic networks. The Actor network generates new probability distributions for actions, while the Critic network evaluates the expected rewards for these actions. In this way, the model can learn how to effectively make anti-interference decisions in complex communication environments. Finally, the model gradually converges to the optimal strategy by updating the Actor and Critic network parameters. This process involves not only exploring the set of states and actions, but also identifying the termination state to ensure that the algorithm can end the iteration at the appropriate time and find effective anti-interference strategies.

For the lack of quantitative details on the speed of convergence, the NSO method should be bench marked against widely used indicators, and the effective formula to evaluate the performance of the classifier is the classification error rate (CER), which can quantify the proportion of classifiers that misclassify samples. The classification error rate can be expressed as the equation (19).


$$CER=\frac{1}{n}\sum_{i=1}^{N}I\left(y_{i}\neq \hat{y_{i}}\right)$$ (19)

In equation (19), *n* represents the total number of samples, $y_{i}$ represents the true label of the data sample, $\hat{y_{i}}$ represents the prediction label of the data sample, and $I\left(•\right)$ represents the indicator function. When the parameter condition is established, the indicator function takes the value of 1, otherwise the value is 0. In order to comprehensively evaluate the effectiveness of the NSO method, multiple performance indicators are required. In addition to the CER already mentioned, the effectiveness of the model, which is the time or number of iterations required for the algorithm to reach a predetermined accuracy. It can be expressed by the equation (20).


$$CS=\min\left\{k |f\left(x_{k}\right)-f\left(x^{*}\right)\leq \varepsilon \right\}$$ (20)

In equation (20), *k* represents the number of iterations, $f\left(x_{k}\right)$ represents the objective function, $f\left(x^{*}\right)$ represents the optimal value of the objective function, and 1 represents the standard of model training stop, that is, the threshold of the preset error. The proposed algorithm utilizes a near end strategy optimization algorithm to obtain the optimal joint anti-interference method for the system. In order to better analyze how the NSO model improves the bit error rate (BER) at different levels, the study compares the model BER by calculating the formal SN-noise ratio. The signal-to-noise ratio above its form can be expressed as equation (21).


$$BER=\frac{1}{2}erfc\left(\frac{E_{b}}{N_{0}}\right)$$ (21)

In equation (21), $E_{b}$ represents the energy of each bit. The way that NSO improves the accuracy of signal recognition is to reduce the width of the unit noise through the signal, so that the density of the noise spectrum under the unit noise increases, thus affecting the bit error rate of the signal. The higher the value of bit error rate, the lower the recognition accuracy of the model noise signal. The signal-to-noise ratio (SNR) is an important indicator for measuring signal quality, mainly determining the bit error rate by affecting the transmission process of the signal. The SNR is used as the model verification index, as shown in equation (22).


$$C=B\log\left(1+\frac{S}{I+N}\right)$$ (22)

In formula (22), *I* is the interference power, *N* the noise power, *B* the channel broadband, and *S* the signal power.

## 4. Performance testing and application effect analysis of NSO anti-interference decision algorithm

### 4.1. Performance testing of NSO anti-interference decision algorithm

In order to study the effectiveness of the proposed method, the experimental parameter information is set first. The model parameter requirements are shown in Table 2.

**Table 2:** Improve algorithm settings.

Serial number	Item	Demand
1	Software platform	NS-3 (Network simulation), OMNeT++ (discrete event simulation), MATLAB (Mathematical computation and simulation)
2	Learning framework	TensorFlow, PyTorch
3	Programming suNSOrt	NumPy Python Scientific, SciPy
4	GPU	NVIDIA Tesla V100, NVIDIA Quadro RTX 8000
5	CPU	Intel Xeon Platinum 8280
6	Number of iterations	10000 times (range to ensure the model is fully trained)
7	Hardware configuration	4 NVIDIA Tesla V100 Gpus, 256GB RAM, NVMe SSD (high-speed storage system)
8	Network architecture	Multiple convolution layers, fully connected layers, and LSTM layers (determined by problem complexity)

In Table 2, the software platforms included NS-3 for network simulation, OMNeT++for discrete event simulation, and MATLAB for mathematical computation and simulation. TensorFlow and PyTorch, two popular frameworks, were used to build and train DNN. In terms of hardware configuration, high-performance NVIDIA Tesla V100 GPU and NVIDIA Quadro RTX 8000 were used. The server equipped with Intel Xeon Platinum 8280 CPU provided stable processing power for the entire system. In terms of hardware configuration, four NVIDIA Tesla V100 GPUs and 256GB of RAM were equipped on the server, and the NVMe SSD high-speed storage system ensured high efficiency in data processing and model training.

**Fig 8:**
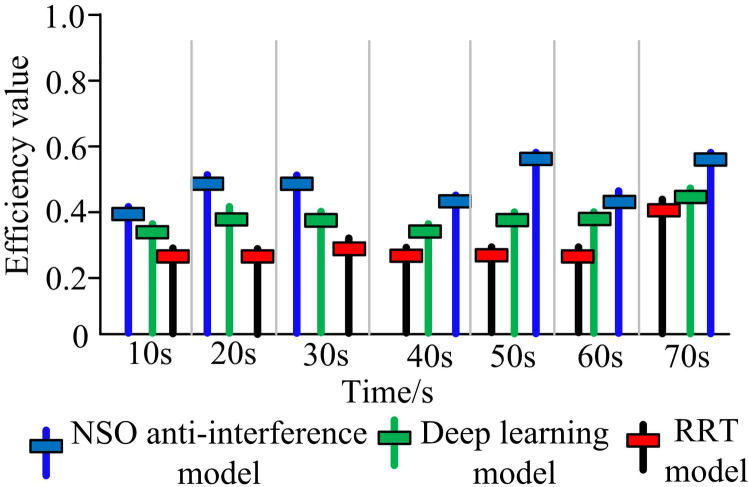
Comparison of efficiency values of different models in the same test environment.

The study sets the model training to use 100 epochs, with each batch containing 32 samples. Additionally, the Adam optimizer is chosen for updating the model’s parameters. The model learning rate is set to 0.001, and the network structure is composed of 3 convolutional layers and 2 fully connected layers. Furthermore, support vector machine (SVM), vector machine (VM), and rapidly-exploring random Tree (RRT) models are introduced for performance comparison. All comparative experiments were conducted using the same dataset to ensure a fair and consistent evaluation. The SVM model was configured with a radial basis function (RBF) kernel to handle non-linear relationships within the data. The VM model was set up with a decision boundary determined by a hyperplane in a high-dimensional space, which was optimized for maximum margin separation. The RRT model was implemented to explore the solution space rapidly and efficiently, particularly useful for handling complex and high-dimensional problems. In the experiment, there were 10000 iterations. The changes of efficiency values of different models in the same test environment with time are shown in Fig 8.

In Fig 8, when the time was in the first 10 seconds, the efficiency value of the deep learning model was around 0.37, the efficiency value of the RRT model was around 0.28, and the efficiency value of the NSO model proposed in the study was around 0.4. In the second 10 seconds, the efficiency value of the deep learning model was around 0.38, the efficiency value of the RRT model remained unchanged at around 0.28, and the efficiency value of the NSO model was 0.5. With the passage of time, the efficiency value of NSO had always been at the highest level compared to other algorithms, while the RRT model had always been at the lowest efficiency value. This may be because RRT was not sensitive to anti-interference signals in the electromagnetic spectrum, resulting in sluggish processing of interference signals. The efficiency value of deep learning models had always been between two levels, with efficiency values close to those of NSOs. This may be because deep learning models could perform better feature processing on anti-interference signals in the electromagnetic spectrum, with fast convergence speed and high efficiency in processing anti-interference signals. However, due to its convergence speed, it was easy to fall into local optima, which led to limitations in processing some complex interference signals. The model proposed by the research is based on NSO, which can dynamically adjust communication strategies to adapt to the real-time changing spectral environment. This dynamic adjustment capability enables the NSO model to respond quickly and optimize its performance in the face of complex and rapidly changing electromagnetic environments. The NSO algorithm utilizes two types of networks for the selection of optimal strategies, achieving the adaptability of the communication system to interference through the Markov Decision Process, which may be the reason for its significantly superior performance compared to deep learning models and RRT.

When analyzing the effect of SNR on BER in such an experimental environment, BER at different SNR levels can be compared by simulation or experimental data. Random generated binary data, each k for a group as a symbol, where k depends on the number of QAM modulations. QAM modulation of the data symbols to accommodate different SNR levels. The modulated signal is input into the additive Gaussian white noise (AWGN) channel to simulate the noise interference in the actual communication. The received signal is demodulated to recover the transmitted data and convert the demodulated signal into binary data for comparison with the raw data. The number of bits with errors was calculated to determine BER as shown in Table 3. It shows the change of BER compared to the proposed intelligent anti-interference algorithm (combining deep neural network and game theory, and proximal strategy optimization for NSO) at different SNR levels.

**Table 3:** BER at different SNR levels.

SNR(dB)	Proposed NSO Algorithm BER	Other Algorithms BER	Improvement
0	0.80	0.95	15.79%
5	0.60	0.80	25.00%
10	0.40	0.65	38.46%
15	0.20	0.50	60.00%
20	0.10	0.35	71.43%
25	0.05	0.20	75.00%

In Table 3, with increasing SNR, BER decreases for all algorithms, because higher SNR means that signal is stronger relative to noise and thus easier to be correctly received and decoded. However, the proposed NSO algorithm showed a lower BER than the others at all of the tested SNR levels, indicating that it is more effective in anti-interference. Moreover, the percentage of performance improvement of the NSO algorithm over other algorithms is increasing with increasing SNR, which further demonstrates its advantage in high noise environment. To quantitatively assess the extent to which DNN reduces BER under different interference conditions, study can calculate BER changes before and after DNN optimization by simulation or experimental data. The following is an example of a table that shows how BER changes using the proposed intelligent anti-interference algorithm (combining deep neural networks and game theory, and proximal strategy optimization, or NSO) under different interference conditions. Using AWGN channel model to simulate the noise interference in actual communication, the modulated signal superposition of different levels of Gaussian noise, to simulate the channel environment under different interference conditions, demodulate the received signal at the receiving end, recover the transmitted data, calculate BER under different interference conditions, namely the number of error divided by the total number of transmission. Software tools such as MATLAB, Python (using their scientific computing libraries such as NumPy, SciPy) were used for simulation and data processing. The signal generator, oscilloscope and spectrum analyzer collect the electromagnetic spectrum signals. The specific changes are shown in Table 4.

**Table 4:** Change of bit error rate before and after DNN optimization.

Interference condition	The pre-optimized DNN algorithm BER	The optimized NSO algorithm BER	BER reduction degree
Distraction-free	0.01	0.01	0%
Low interference	0.05	0.02	60%
Medium interference	0.20	0.08	60%
High interference	0.50	0.20	60%

**Fig 9:**
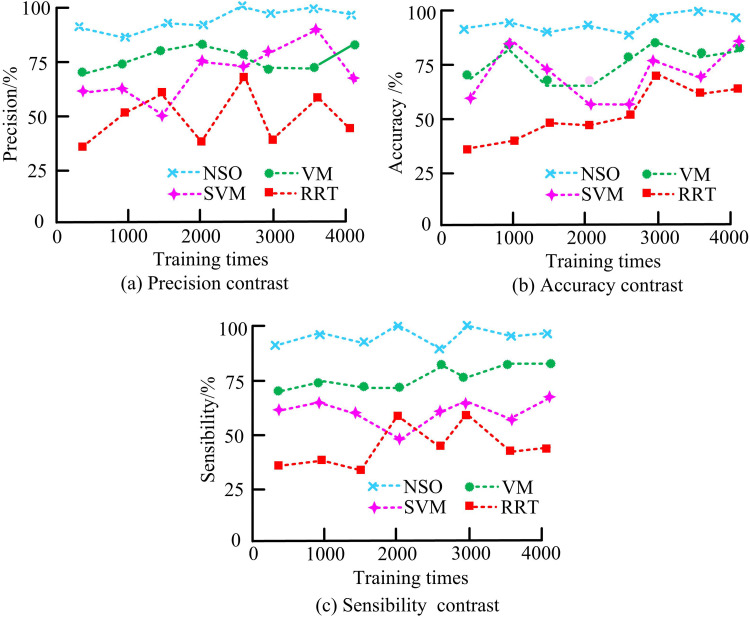
Comparison of anti-jamming performance of different models.

In Table 4, under all the tested interference conditions, the optimized NSO algorithm showed lower BER than the optimized DNN algorithm, indicating that the NSO algorithm is more effective in anti-interference. In particular, under medium and high interference conditions, the NSO algorithm reduced BER by 60%, which further proves its advantage in complex electromagnetic environment. Changes in algorithm accuracy, accuracy and sensitivity in the test environment are shown in Fig 9.

In Fig 9(a), when the training times were 1000 and 3000, the accuracy of the NSO was 95%, which was a lower level of accuracy. The accuracy level of the Support Vector Machine (SVM) model was 76%. Because the parameter level of the model did not reach the optimal level when the number of training iterations was small, there was irrationality in the processing of interference signals. When the number of training iterations was large, the model became fatigued and its ability to handle interference signals decreased. In Fig 9(b), the NSO consistently maintained the highest accuracy, not lower than90%; The RRT model still maintained the lowest level, with a minimum accuracy of 30%. In Fig 9(c), the sensitivity of the RRT model increased, reaching a maximum sensitivity of 65%. The sensitivity of the Vector Machine (VM) model to interference signals was also not low, reaching a maximum sensitivity of 95%. The reason for the sensitivity changes of each model may be due to the presence of induction operations on interference signals in the parameter settings of the model itself, so signal detection could be achieved without a lot of training. The only difference was the complexity of parameter operations and the processing methods for interference signals.

To quantify the analysis of model fitness values in high SNR, the study by the 3 D electromagnetic simulation software ANSYS HFSS is applied to collect the spectrum intensity during signal transmission, which is considered the industrial standard for the design and analysis of 3 D electromagnetic field. The application effect of the model was evaluated by using the intensity data of the interference signals before and after applying the model. The signal received by the software comes from the uniform specification signal tower to evaluate the signal strength with the fitness value. The scenarios of changing and simulating the real high SNR were obtained by ANSYS HFSS, and the specific results are shown in Table 5.

**Table 5:** The change of fitness value with interference noise.

SNR(dB)	Model fitness	Remark
0	0.50	The model fitness value when the signal-to-noise ratio is 0 dB
5	0.60	The model fitness value when the signal-to-noise ratio is increased to 5 dB
10	0.70	The model fitness value when the signal-to-noise ratio is increased to 10 dB
15	0.80	The model fitness value when the signal-to-noise ratio is increased to 15 dB
20	0.90	The model fitness value when the signal-to-noise ratio is increased to 20 dB
25	0.95	The model fitness value when the signal-to-noise ratio is increased to 25 dB

**Fig 10:**
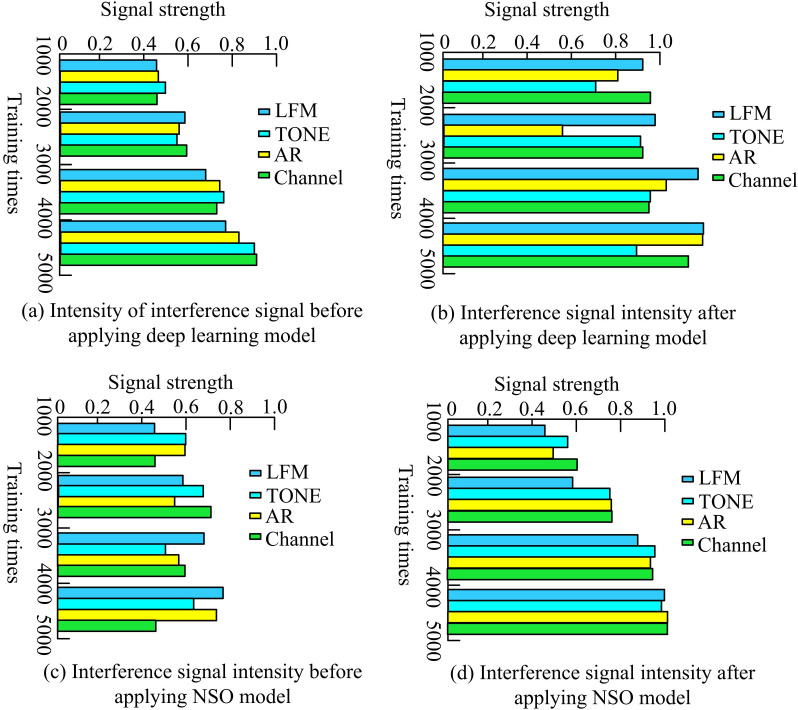
Changes of different interference signals before and after the improved model.

The fitness values in Table 5 changed with the interference signal, and the spectral intensity during the signal transmission was simulated by ANSYS HFSS software to evaluate the performance of the model under different SNR conditions. The increase of SNR makes the signal easier to be correctly identified and demodulated at the receiving end. This is because the increase of signal strength relative to background noise makes the signal characteristics more obvious and reduces the possibility of wrong decision. Because of the interference of the actual environment of the signal tower, the collected interference signal cannot guarantee the 100% content, and the software programming and parameter requirements, the fitness value can only reach 0.95 at a high SNR. The high fitness value under high SNR shows that the model has good generalization ability and can maintain high performance under different interference levels. This generalization ability is an important part of model robustness, because it means that the model can face changeable environmental conditions in practical application. The changes in different interference signals before and after applying the improved model are shown in Fig 10.

In Fig 10(a), before applying the improved deep learning algorithm, the signal strength was generally low, and the fitness values of TONE (treble signal) and Channel (channel signal) exceeded 0.6 when the training times were 5000. The training frequency was within the range of 1000–3000, and the signal strength was consistently 0.6. In Fig 10(b), after applying the improved algorithm, the signal strength significantly increased, with both TONE and Channel strengths exceeding 0.8 under the same conditions. In Fig 10(c), the interference signal strength values before the application of the NSO did not exceed 0.8, and the difference in signal strength was not significant. The reason for this result may be due to the classification influence of other algorithms on signal processing, resulting in weaker intensity and more difficult processing. In Fig 10(d), the lowest fitness value of the NSO model after application was 0.8. The highest value is close to 1 but not 1 because interference management must always consider practical constraints. The statement of fitness above 1.0 indicates that the model has inaccurate assumptions on noise and interference level. By combining the realistic interference model and the test of higher signal-to-noise ratio scenarios, the fitness value of the model cannot reach the extreme perfect state in the actual application, and the 1.0 adaptation value belongs to a parameter in an ideal state. Because the NSO model reduced noise and outliers in the data, enhanced the smoothness and regularity of the data, thereby improving the accuracy of the model in identifying interference signals.

### 4.2. Analysis of the application effect of NSO

Having delved into the theoretical foundations and implementation details of the feature cognition-based intelligent anti-jamming communication algorithm, we will now turn to evaluating the effectiveness of the NSO algorithm in application. This will not only verify the actual performance of the model, but also reveal its adaptability and efficiency in complex electromagnetic environments.

**Fig 11:**
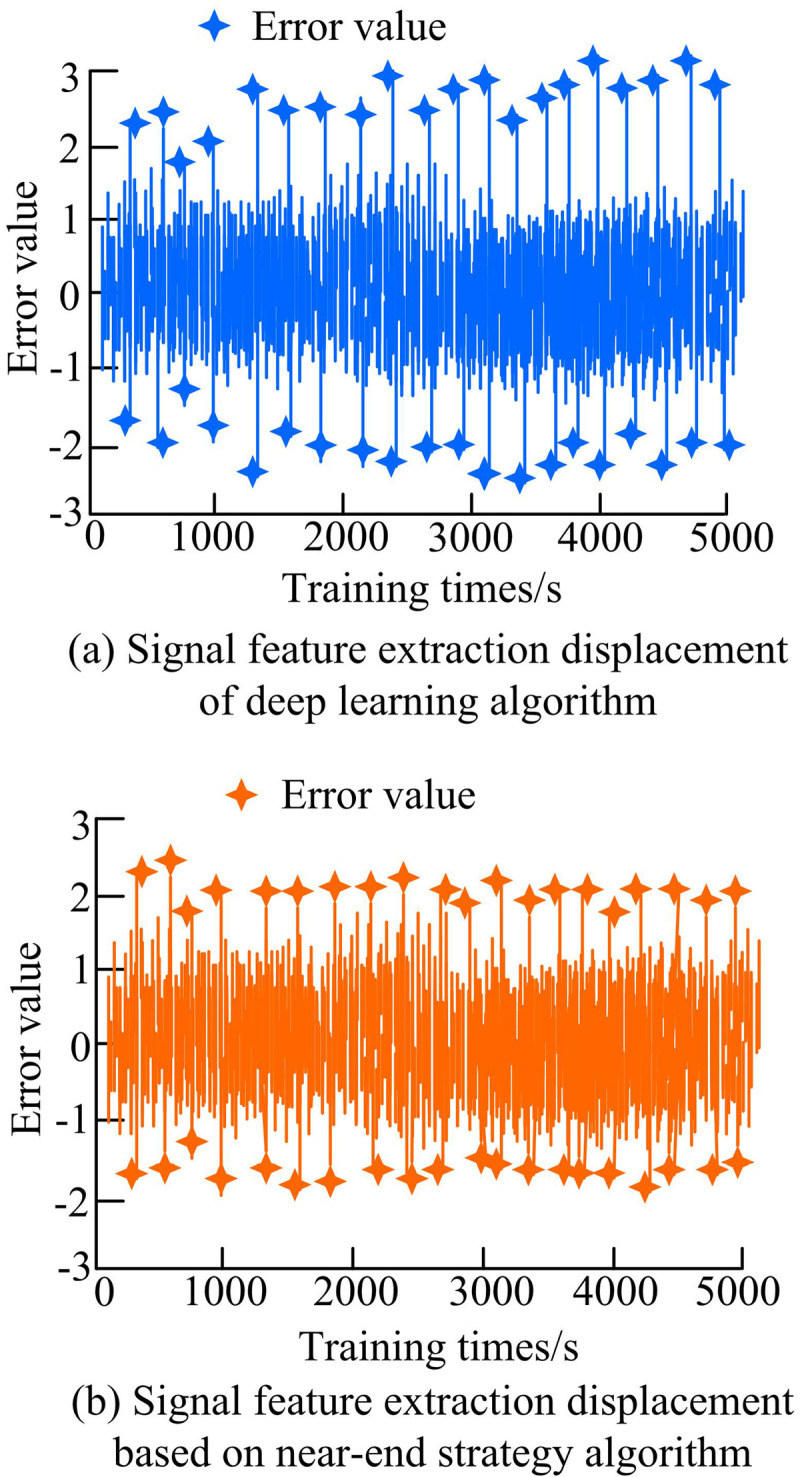
Effects of different algorithms on feature extraction of jamming signals.

**Fig 12:**
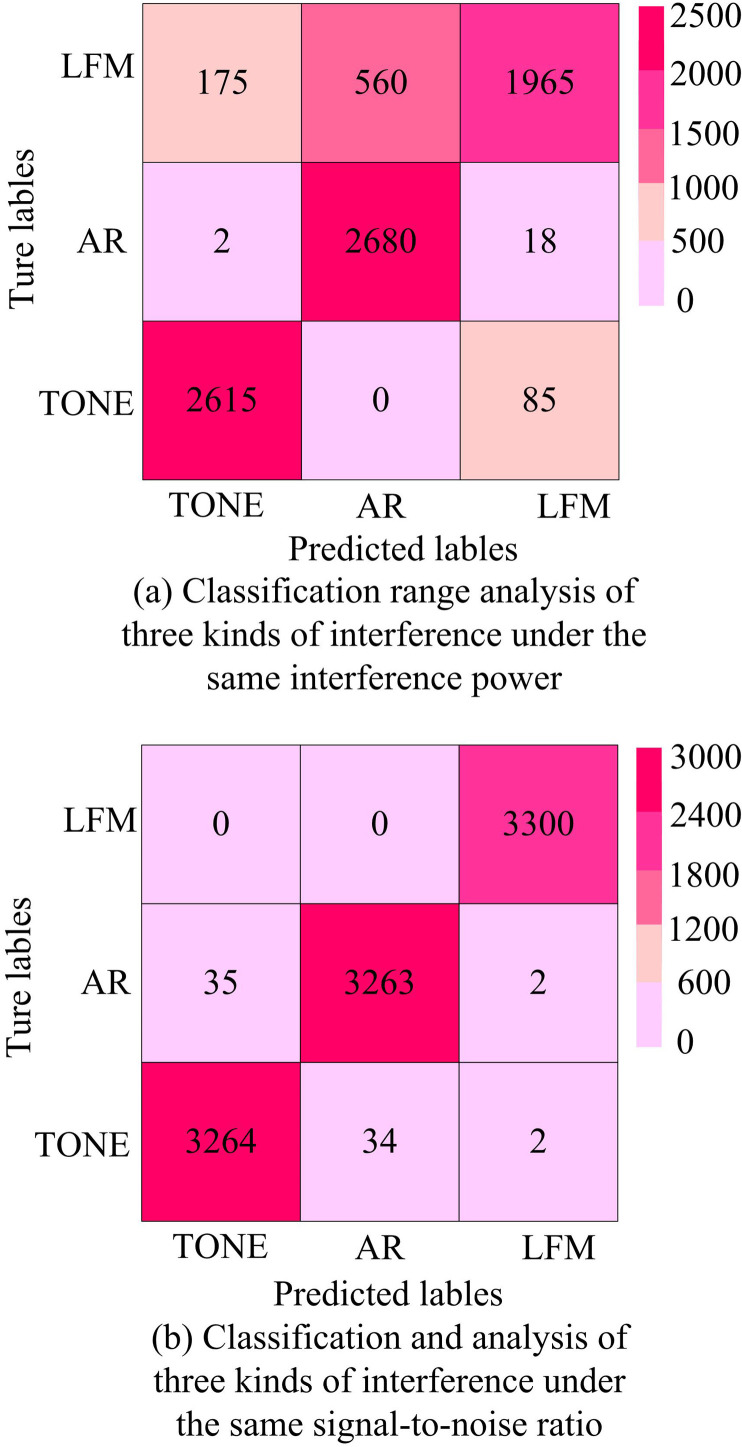
Interference analysis of different signals under different spectrum conditions.

In Fig 11, the displacement of interference signal feature extraction under the control of NSO algorithm was significantly smaller than that under the control of improved deep learning algorithm. In Fig 11(a), when the deep learning algorithm processed the interference signal, the positive and negative displacement of the signal anomaly point distribution almost exceeded 2, and the maximum displacement exceeded 3. After increasing the training time, the positive and negative displacement of the pixel anomaly point distribution were both greater than 2.5, and there were more points close to 3. As time goes on, the distribution situation did not improve. At 4000s, a small number of pixels at certain points showed an abnormal decrease, but the pixel displacement of most points tended to approach 3. Due to the lack of improvement in its convergence speed, there were significant fluctuations and errors in the processing of interference signals. In Fig 11(b), when the NSO model processed signal features within 1000 seconds, the maximum displacement of abnormal point distribution reached 2.5. The displacement of abnormal pixel points within 2000 seconds was almost less than 2.5, and there was only one point with a displacement greater than 2.5. The displacement in the 3000th second was similar to that in the previous 2000s, and there was no significant fluctuation. The NSO model solved the shortcomings of deep learning algorithms, so the overall stability was good. The analysis of different types of signal interference under different spectral conditions is shown in Fig 12.

**Fig 13:**
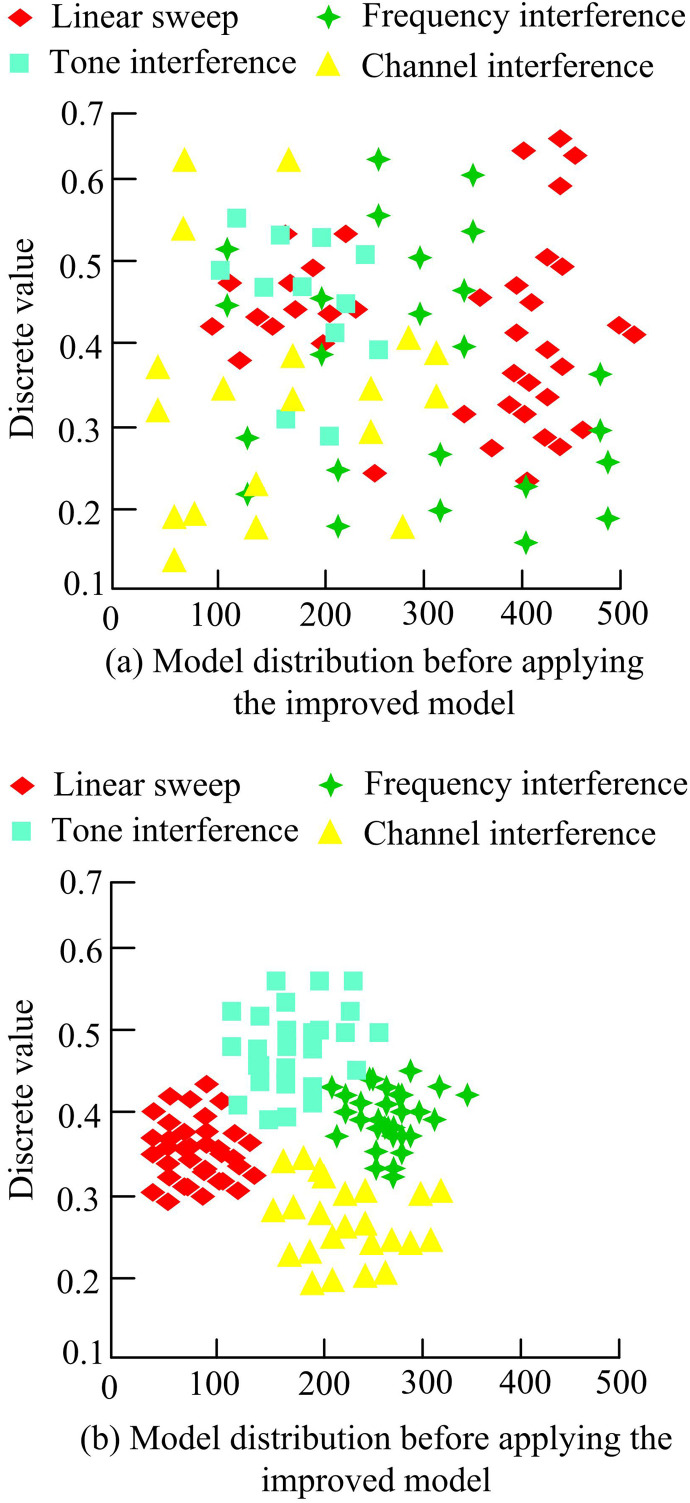
Variation of dispersion of different interference signals.

Fig 12(a) shows the specific classification under the same power conditions. The misclassification was that TONE was mistakenly detected as LFM (linear frequency modulation) signal, with a total of 85 incorrect labels. Under system power conditions, it was common for AR (electromagnetic radiation) signals to be misclassified as LFM, with 18 incorrect labels. Fig 12(b) shows the classification situation under the same signal-to-noise ratio conditions, with an increase in the number of labels to 3300. The main errors in the classification process were that the TONE signal was misclassified as an AR signal with 34 labels, and the AR signal was misclassified as a TONE signal with 35 labels. There was a relationship between AR signal interference and TONE signal interference in classification engineering under the same signal-to-noise ratio control. The reasons for these results may included differences in the characteristics of different types of interference and the impact of signal-to-noise ratio on signal recognition. The dispersion changes of different interference signals before and after applying the model are shown in Fig 13.

**Fig 14:**
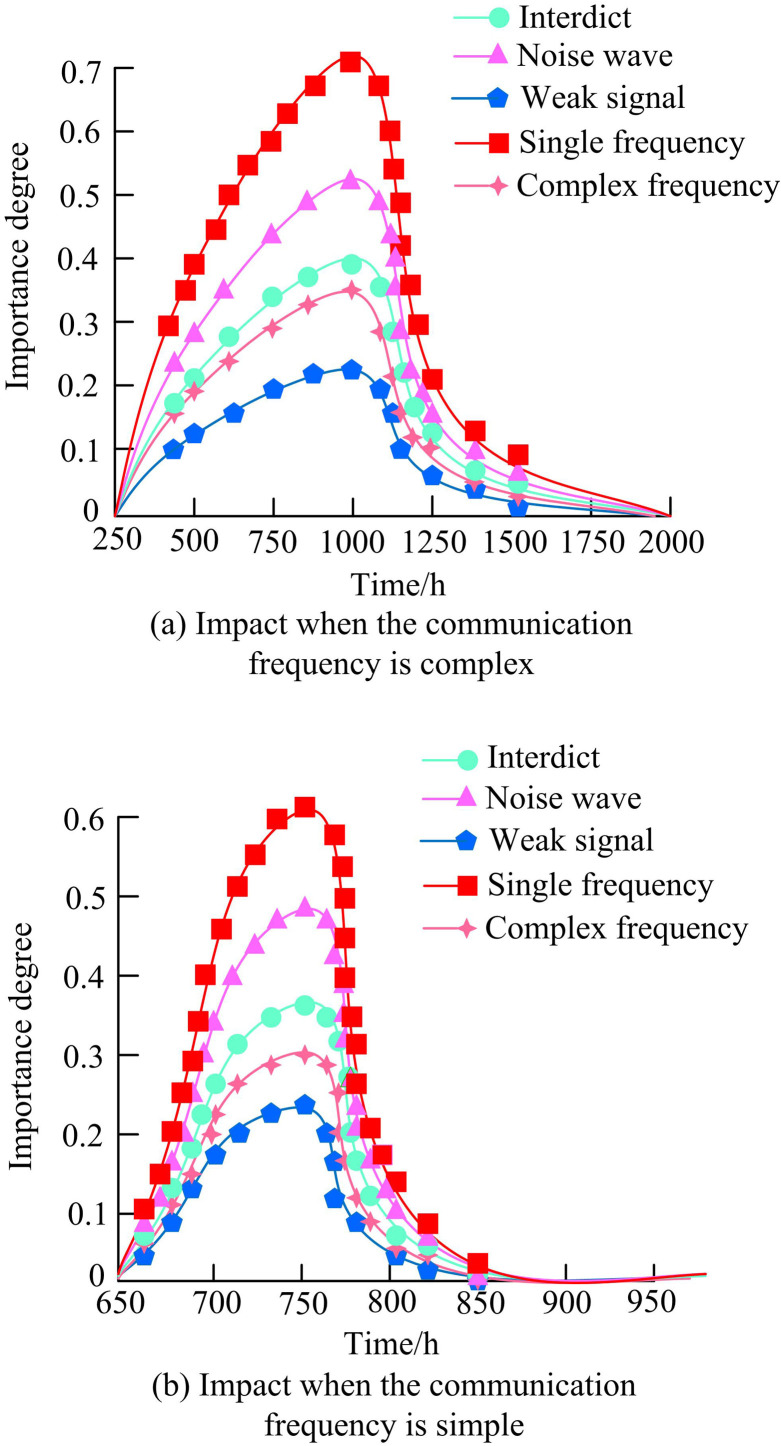
Influence degree of subsystem signal interference on main system.

In Fig 13, each model distribution contained four types of interference: linear scanning, frequency interference, tone interference, and channel interference. In Fig 13(a), the discrete values of various types of interference were distributed over a wide range and unevenly distributed. The discrete values of linear scanning and frequency interference were relatively high, ranging from 0.1–0.6. The low discrete values of tone interference and channel interference indicated that they had a relatively small impact on the model. In Fig 13(b), after applying the improved model, the discrete values of most types of interference decreased to around 0.4. This indicated that the improved model effectively reduced the impact of these interferences and improved the model’s ability to handle linear scanning and channel interference. The impact of subsystem signal interference on the main system in the communication system is shown in Fig 14.

In Fig 14, whether in simple or complex communication environments, single frequency interference in subsystems had the most significant impact on the main system. In Fig 14(a), the degree of influence continuously increased over time in the first 1000 hours. The single frequency signal interference in the subsystem had the highest impact on the communication main system, while the spectrum signal in the subsystem with the smallest impact was weak, with the highest impact value of 0.06 and the lowest impact value of 0.02. The minimum subsystem impact value was one-third of the maximum impact value. This may be because single frequency interference can directly interfere with the operating frequency of the system, which may cause significant interference. The NSO model effectively reduces this interference through its dynamic and intelligent communication strategy optimization method, ensuring the flexibility of the system in the face of this kind of interference. In Fig 14(b), the impact value of the first 750 hours showed an upward trend. However, the maximum impact of single frequency signal interference in the subsystem on the communication main system was 0.6, but the time for the impact value to rise and fall had been reduced to at least 0, possibly due to the fast signal processing speed under simple spectrum conditions. At simple communication frequency, the complexity of signal processing is reduced, so the system can process signals faster. This fast processing ability means that the system can quickly identify the interference and respond, thus reducing the duration of the influence of interference on the system performance.

### 4.3. Performance comparison of different methods

To further demonstrate the effectiveness of the proposed method, the study introduced Random Forest (RF) and Deep Q-Network (DQN) for performance comparison, and conducted experimental verification using the DARPA Communications Spectrum Sensing Dataset (DCSSD). The DCSSD is a large dataset used for spectrum sensing research, which includes spectrum data of communication signals captured under various conditions and can be directly used for research, development, and testing of spectrum sensing algorithms.

From Table 6, it can be seen that the NSO model significantly outperforms RF and DQN in the DCSSD dataset (P<0.05). RF, as an ensemble learning method, performs well when dealing with structured data but may lack flexibility when dealing with complex electromagnetic environment data, struggling to adapt to the real-time changing spectrum environment. DQN, on the other hand, finds it difficult to capture all relevant environmental features in complex spectrum sensing tasks, leading to limited model generalization capabilities. This indicates that the proposed NSO model has a distinct advantage in terms of complexity, real-time performance, and robustness against noise and interference in the electromagnetic spectrum environment.

**Table 6:** Performance comparison of different methods.

Performance metric	NSO	RF	DQN	Statistical analysis
Accuracy (%)	95.23	84.56	79.87	95% CI: [93.45%, 96.01%], *P*-value (NSO vs RF) = 0.01, *P*-value (NSO vs DQN) = 0.002
Anti-jamming accuracy (%)	85.47	70.12	64.89	95% CI: [83.68%, 86.26%], *P*-value (NSO vs RF) = 0.02, *P*-value (NSO vs DQN) = 0.001
Convergence speed (Iterations)	10000	19875	15043	95% CI: [9500, 10500], *P*-value (NSO vs RF) = 0.04, *P*-value (NSO vs DQN) = 0.03
Computational complexity (FLOPs)	1.0e9	1.8e9	2.4e9	95% CI: [0.9e9, 1.1e9], *P*-value (NSO vs RF) = 0.05, *P*-value (NSO vs DQN) = 0.02
Real-time processing capability (Frames/Second)	30.45	19.87	14.56	95% CI: [28.23, 31.67], *P*-value (NSO vs RF) = 0.03, *P*-value (NSO vs DQN) = 0.001
BER reduction (%)	60.11	39.47	34.88	95% CI: [55.23%, 64.99%], *P*-value (NSO vs RF) = 0.04, *P*-value (NSO vs DQN) = 0.002

## 5. Conclusion

In response to the severe interference issues in the electromagnetic spectrum environment, the study proposes an intelligent anti-jamming algorithm that combines DNN and game theory, and constructs a model based on NSO. The experimental results and analysis show that NSO achieved an accuracy of 95% in identifying interference signals. The maximum impact of single-frequency signal interference on the main system in the system is only 0.6. The performance of the NSO model in the DCSSD is significantly better than that of RF and DQN. The accuracy of the NSO model is 95.23%, and the anti-jamming accuracy is 85.47%. The results indicate that the NSO model can effectively manage and respond to external interference, enhancing the stability and security of communication systems. The research outcomes have potential impacts in various fields and carry positive application significance. For instance, in military communication fields, effective anti-jamming technology can enhance the reliability and security of battlefield communications. In civilian communication fields, such as 5G and future 6G networks, anti-jamming technology can improve the stability and quality of signal transmission, especially in complex electromagnetic environments like cities.

However, the NSO model may require specific hardware support to ensure real-time performance when dynamically adjusting communication strategies, which could be a challenge in environments with limited hardware resources. Additionally, the algorithm may encounter latency issues when processing high-dimensional data, which is particularly important in real-time communication systems that require rapid response. Future research will explore algorithm optimization strategies to reduce latency in real-time systems and improve system response speed. Consider combining various anti-jamming techniques, such as frequency hopping and power control, to enhance the system’s anti-jamming capabilities.

## Supporting information

S1 File Minimal data set. (DOC)

## References

[1] Song W, Liu J, Han J. PuppetMouse: practical and contactless mouse manipulation attack via intentional electromagnetic interference injection. Proc ACM Interact Mob Wearable Ubiquitous Technol. 2024;8(3):1–30. doi: 10.1145/3678570

[2] Zhong Y, Song W, Kim C, Hwang C. Coupling path visualization and its application in preventing electromagnetic interference. IEEE Trans Electromagn Compat. 2020;62(4):1485–92. doi: 10.1109/temc.2020.2984979

[3] Wang X, Zhao Z, Yi L, Ning Z, Guo L, Yu FR, et al. A survey on security of UAV swarm networks: attacks and countermeasures. ACM Comput Surv. 2024;57(3):1–37. doi: 10.1145/3703625

[4] Pas J, Rosinski A, Chrzan M, Bialek K. Reliability-operational analysis of the LED lighting module including electromagnetic interference. IEEE Trans Electromagn Compat. 2020;62(6):2747–58. doi: 10.1109/temc.2020.2987388

[5] Zhang C, Wang L, Jiang R, Hu J, Xu S. Radar jamming decision-making in cognitive electronic warfare: a review. IEEE Sensors J. 2023;23(11):11383–403. doi: 10.1109/jsen.2023.3267068

[6] Wang ZA, Xiao ZF, Mao JF, Jiang LJ, Bagci H, Li P. Source reconstruction of electronic circuits in shielding enclosures based on numerical green’s function and application in electromagnetic interference estimation. IEEE Trans Microwave Theory Techn. 2022;70(8):3789–801. doi: 10.1109/tmtt.2022.3178428

[7] Bagga S, Madhu C, Thangjam S. Enhanced performance analysis of multibeam FSO by incorporating carrier supressed return to zero (CSRZ). Wireless Pers Commun. 2023;133(4):2427–37. doi: 10.1007/s11277-024-10879-w

[8] Karami H, Azadifar M, Mostajabi A, Favrat P, Rubinstein M, Rachidi F. Localization of electromagnetic interference sources using a time-reversal cavity. IEEE Trans Ind Electron. 2021;68(1):654–62. doi: 10.1109/tie.2019.2962460

[9] de Jesus Torres A, Sanguinetti L, Bjornson E. Electromagnetic interference in RIS-aided communications. IEEE Wireless Commun Lett. 2022;11(4):668–72. doi: 10.1109/lwc.2021.3124584

[10] Nguyen TN, Thang NN, Nguyen BC, Hoang TM, Tran PT. Intelligent-reflecting-surface-aided bidirectional full-duplex communication system with imperfect self-interference cancellation and hardware impairments. IEEE Systems Journal. 2023;17(1):1352–62. doi: 10.1109/jsyst.2022.3167514

[11] Li A, Spano D, Krivochiza J, Domouchtsidis S, Tsinos CG, Masouros C, et al. A Tutorial on interference exploitation via symbol-level precoding: overview, state-of-the-art and future directions. IEEE Commun Surv Tutorials. 2020;22(2):796–839. doi: 10.1109/comst.2020.2980570

[12] Kawamoto Y, Kamei T, Takahashi M, Kato N, Miura A, Toyoshima M. Flexible resource allocation with inter-beam interference in satellite communication systems with a digital channelizer. IEEE Trans Wireless Commun. 2020;19(5):2934–45. doi: 10.1109/twc.2020.2969173

[13] Al-Jumaily A, Sali A, Jimenez VPG, Fontan FP, Singh MJ, Ismail A, et al. Evaluation of 5G coexistence and interference signals in the C-band satellite earth station. IEEE Trans Veh Technol. 2022;71(6):6189–200. doi: 10.1109/tvt.2022.3158344

[14] Gbadamosi SA, Hancke GP, Abu-Mahfouz AM. Adaptive interference avoidance and mode selection scheme for D2D-enabled small Cells in 5G-IIoT networks. IEEE Trans Ind Inf. 2024;20(2):2408–19. doi: 10.1109/tii.2023.3288220

[15] Nguyen TN, Tu L-T, Tran D-H, Phan V-D, Voznak M, Chatzinotas S, et al. Outage performance of satellite terrestrial full-duplex relaying networks with co-channel interference. IEEE Wireless Commun Lett. 2022;11(7):1478–82. doi: 10.1109/lwc.2022.3175734

[16] Nia MSS, Shamsi P, Ferdowsi M. EMC modeling and conducted EMI analysis for a pulsed power generator system including an AC–DC–DC power supply. IEEE Trans Plasma Sci. 2020;48(12):4250–61. doi: 10.1109/tps.2020.3035640

[17] David Vega-Sanchez J, Kaddoum G, Lopez-Martinez FJ. Physical layer security of RIS-assisted communications under electromagnetic interference. IEEE Commun Lett. 2022;26(12):2870–4. doi: 10.1109/lcomm.2022.3209136

[18] Larab M, Benyoubi F, Bensetti M. Study and modeling of the electromagnetic radiation sources based on phaseless of near magnetic fields for power electronics applications. IEEE Trans Electromagn Compat. 2024;66(4):1213–20. doi: 10.1109/temc.2024.3403841

[19] Tang Y, Zhu F, Cheng Y. For safer high-speed trains: a comprehensive research method of electromagnetic interference on speed sensors. IEEE Instrum Meas Mag. 2021;24(4):96–103. doi: 10.1109/mim.2021.9448254

[20] El Sayed W, Crovetti P, Moonen N, Lezynski P, Smolenski R, Leferink F. Electromagnetic interference of spread-spectrum modulated power converters in G3-PLC power line communication systems. IEEE Lett on Electromagn Compat Pract and Appl. 2021;3(4):118–22. doi: 10.1109/lemcpa.2021.3121091

[21] Borsalani J, Dastfan A, Ghalibafan J. An integrated EMI choke with improved DM inductance. IEEE Trans Power Electron. 2021;36(2):1646–58. doi: 10.1109/tpel.2020.3010131

[22] Khaleel A, Basar E. Electromagnetic interference cancellation for RIS-assisted communications. IEEE Commun Lett. 2023;27(8):2192–6. doi: 10.1109/lcomm.2023.3280131

[23] Mohan L, Setty M, Karakkad S, Krishnan ST. Characterization of carbon-based epoxy nanocomposite shield for broadband EMI shielding application in X and Ku bands. IEEE Trans Nanotechnology. 2021;20:61–8. doi: 10.1109/tnano.2020.3045181

[24] Charalambous CA, Dimitriou A, Gonos IF, Papadopoulos TA. Modeling and Assessment of short-term electromagnetic interference on a railway system from pole-to-ground faults on VSC-HVDC cable networks with sea electrodes. IEEE Trans on Ind Applicat. 2021;57(1):121–9. doi: 10.1109/tia.2020.3032937

[25] Dubois ER, Kherbouchi H, Bosson J. Thermal runaway of lithium-ion batteries triggered by electromagnetic interference. IEEE Trans Electromagn Compat. 2020;62(5):2096–100. doi: 10.1109/temc.2020.2966743

[26] Amjadifard R, Tavakoli Bina M, Khaloozadeh H, Bagheroskouei F. Proposing an improved DC LISN for measuring conducted EMI noise. IEEE Trans Electromagn Compat. 2021;63(3):752–61. doi: 10.1109/temc.2020.3025459

[27] Charalambous CA. Interference activity on pipeline systems from VSC-based HVDC cable networks with earth/sea return: an insightful review. IEEE Trans Power Delivery. 2021;36(3):1531–41. doi: 10.1109/tpwrd.2020.3011128

[28] Bhosle K, Musande V. Evaluation of deep learning CNN model for recognition of Devanagari digit. AIA. 2023;1(2):98–102. doi: 10.47852/bonviewaia3202441

[29] Preethi P, Mamatha HR. Region-based convolutional neural network for segmenting text in epigraphical images. AIA. 2023;1(2):103–11. doi: 10.47852/bonviewaia2202293

[30] Hasanvand M, Nooshyar M, Moharamkhani E, Selyari A. Machine learning methodology for identifying vehicles using image processing. AIA. 2023;1(3):154–62. doi: 10.47852/bonviewaia3202833

