# Supplementary material for: Intelligent anti-jamming communication technology with electromagnetic spectrum feature cognition
Source: PLoS One. 2025 Apr 24;20(4):e0319953. doi: 10.1371/journal.pone.0319953 (PMC12021182; doi:10.1371/journal.pone.0319953)
Supplement: S1 File — (DOC) [file pone.0319953.s001.doc]

**Figure 1, no data**

**Figure 2, no data**

**Figure 3, no data**

**Figure 4, no data**

**Figure 5, no data**

**Figure 6, no data**

**Figure 7, no data**

**The data in Figure 8**

| Model | 10s | 20s | 30s | 40s | 50s | 60s | 70s |
| --- | --- | --- | --- | --- | --- | --- | --- |
| NSO | 0.40 | 0.50 | 0.51 | 0.43 | 0.57 | 0.43 | 0.54 |
| Deep learning | 0.37 | 0.38 | 0.38 | 0.32 | 0.38 | 0.40 | 0.44 |
| RRT | 0.28 | 0.28 | 0.30 | 0.29 | 0.29 | 0.29 | 0.41 |

**The data in Figure 9**

| Index | | Model | | | |
| --- | --- | --- | --- | --- | --- |
|  |  | NSO | VM | SVM | RRT |
| Precision contrast | 1000 | 77 | 75 | 64 | 53 |
|  | 2000 | 94 | 81 | 74 | 38 |
|  | 3000 | 77 | 72 | 76 | 38 |
|  | 4000 | 95 | 81 | 72 | 42 |
| Accuracy contrast | 1000 | 88 | 83 | 85 | 38 |
|  | 2000 | 85 | 68 | 63 | 46 |
|  | 3000 | 87 | 82 | 77 | 67 |
|  | 4000 | 90 | 78 | 82 | 64 |
| Sensibility | 1000 | 89 | 74 | 60 | 38 |
|  | 2000 | 95 | 72 | 39 | 65 |
|  | 3000 | 93 | 79 | 65 | 62 |
|  | 4000 | 91 | 81 | 69 | 47 |

The data in Figure 10

| Figure | Training times | LRM | AR | TONE | Channel | Figure | Training times | LRM | AR | TONE | Channel |
| --- | --- | --- | --- | --- | --- | --- | --- | --- | --- | --- | --- |
| (a) | 1000-2000 | 0.46 | 0.47 | 0.50 | 0.47 | (c) | 1000-2000 | 0.47 | 0.60 | 0.59 | 0.48 |
|  | 2000-3000 | 0.59 | 0.56 | 0.54 | 0.60 |  | 2000-3000 | 0.60 | 0.70 | 0.56 | 0.74 |
|  | 3000-4000 | 0.67 | 0.74 | 0.78 | 0.73 |  | 3000-4000 | 0.72 | 0.50 | 0.53 | 0.58 |
|  | 4000-5000 | 0.81 | 0.85 | 0.91 | 0.94 |  | 4000-5000 | 0.80 | 0.66 | 0.78 | 0.48 |
| (b) | 1000-2000 | 0.93 | 0.81 | 0.70 | 0.95 | (d) | 1000-2000 | 0.46 | 0.57 | 0.50 | 0.62 |
|  | 2000-3000 | 0.97 | 0.56 | 0.93 | 0.95 |  | 2000-3000 | 0.59 | 0.76 | 0.81 | 0.77 |
|  | 3000-4000 | 1.12 | 1.03 | 0.95 | 0.93 |  | 3000-4000 | 0.87 | 0.93 | 0.90 | 1.00 |
|  | 4000-5000 | 1.21 | 1.18 | 0.93 | 1.08 |  | 4000-5000 | 1.12 | 1.08 | 1.02 | 1.21 |

**The data in Figure 11**

| Error number | (a) | | (b) | |
| --- | --- | --- | --- | --- |
| 1 | -1.7 | 2.3 | -1.7 | 2.3 |
| 2 | -2.0 | 2.5 | -1.6 | 2.5 |
| 3 | -1.3 | 1.6 | -1.2 | 1.8 |
| 4 | -1.7 | 2.0 | -1.8 | 2.0 |
| 5 | -2.4 | 2.7 | -1.6 | 2.0 |
| 6 | -1.8 | 2.5 | -1.8 | 2.0 |
| 7 | -2.0 | 2.6 | -1.8 | 2.0 |
| 8 | -2.1 | 2.4 | -1.7 | 2.0 |
| 9 | -2.1 | 2.9 | -1.7 | 2.1 |
| 10 | -2.2 | 2.4 | -1.7 | 1.9 |
| 11 | -2.1 | 2.7 | -1.6 | 1.7 |
| 12 | -2.1 | 2.8 | -1.6 | 2.0 |
| 13 | -2.4 | 2.2 | -1.7 | 1.7 |
| 14 | -2.5 | 2.4 | -1.7 | 1.8 |
| 15 | -2.0 | 2.5 | -1.7 | 1.8 |
| 16 | -2.4 | 3.0 | -1.6 | 1.5 |
| 17 | -1.9 | 2.7 | -1.8 | 1.8 |
| 18 | -2.4 | 2.8 | -1.6 | 1.8 |
| 19 | -2.2 | 3.0 | -1.6 | 1.6 |
| 20 | -2.3 | 2.6 | -1.6 | 1.8 |

**The data in Figure 12**

| True lables | (a) | | | (b) | | |
| --- | --- | --- | --- | --- | --- | --- |
|  | TONE | AR | LFM | TONE | AR | LFM |
| LFM | 175 | 560 | 1965 | 0 | 0 | 3300 |
| AR | 2 | 2680 | 18 | 35 | 3263 | 2 |
| TONE | 2615 | 0 | 85 | 3264 | 34 | 2 |

**The data in Figure 13**

| Linear sweep | (a) | | | (b) | | | |
| --- | --- | --- | --- | --- | --- | --- | --- |
|  | Frequency interference | Tone interference | Channel interference | Linear sweep | Frequency interference | Tone interference | Channel interference |
| 0.24 | 0.64 | 0.56 | 0.65 | 030 | 0.32 | 0.40 | 0.19 |
| 0.45 | 0.15 | 0.28 | 0.56 | 0.32 | 0.33 | 0.42 | 0.23 |
| 0.59 | 0.36 | 0.31 | 0.48 | 0.33 | 0.34 | 0.43 | 0.24 |
| 0.67 | 0.57 | 0.40 | 0.36 | 0.34 | 0.35 | 0.45 | 0.25 |
| 0.68 | 0.48 | 0.48 | 0.12 | 0.35 | 0.36 | 0.50 | 0.27 |
| 0.69 | 0.45 | 0.52 | 0.18 | 0.36 | 0.40 | 0.52 | 0.33 |

**The data in Figure 14**

| (a) Impact when the communication frequency is complex | | | | | |
| --- | --- | --- | --- | --- | --- |
| Time/h | Interdict | Single frequency | Noise wave | Complex frequency | Weak signal |
| 500 | 0.020 | 0.035 | 0.027 | 0.019 | 0.013 |
| 750 | 0.035 | 0.055 | 0.043 | 0.029 | 0.018 |
| 1000 | 0.037 | 0.070 | 0.049 | 0.034 | 0.022 |
| 1250 | 0.013 | 0.020 | 0.014 | 0.010 | 0.007 |
| 1500 | 0.005 | 0.010 | 0.009 | 0.004 | 0.002 |
| 1750 | 0.003 | 0.007 | 0.006 | 0.001 | 0.001 |
| 2000 | 0.000 | 0.000 | 0.000 | 0.000 | 0.000 |
| (b) Impact when the communication frequency is simple | | | | | |
| Time/h | Interdict | Single frequency | Noise wave | Complex frequency | Weak signal |
| 700 | 0.027 | 0.046 | 0.034 | 0.027 | 0.016 |
| 750 | 0.035 | 0.060 | 0.048 | 0.030 | 0.025 |
| 800 | 0.006 | 0.014 | 0.012 | 0.005 | 0.004 |
| 850 | 0.000 | 0.003 | 0.001 | 0.000 | 0.000 |
| 900 | 0.000 | 0.000 | 0.000 | 0.000 | 0.000 |
| 950 | 0.000 | 0.000 | 0.000 | 0.000 | 0.000 |
